# Impact of Community Referral on Colonoscopy Quality Metrics in a Veterans Affairs Medical Center

**DOI:** 10.14309/ctg.0000000000000460

**Published:** 2022-01-26

**Authors:** Vincent Petros, Erin Tsambikos, Mohammad Madhoun, William M. Tierney

**Affiliations:** 1 Digestive Diseases and Nutrition Section, Oklahoma University Health Sciences Center, Oklahoma City, Oklahoma, USA;; 2 Oklahoma City VA Medical Center, Oklahoma City, Oklahoma, USA;; 3 Internal Medicine Section, Oklahoma University Health Sciences Center, Oklahoma City, Oklahoma, USA.

## Abstract

The Veterans Access, Choice, and Accountability Act of 2014 expands the number of options veterans have to ensure timely access to high-quality care. There are minimal data currently available analyzing the impact and quality of colonoscopy metrics in veterans receiving procedures within the Department of Veterans' Affairs (VA) vs community settings.

**METHODS:**

All patients at our academic VA medical center who were referred to a community care colonoscopy (CCC) for positive fecal immunochemical testing, colorectal cancer screening, and adenoma surveillance from 2015 to 2018 were identified and matched for sex, age, and year of procedure to patients referred for a VA-based colonoscopy (VAC). Metrics measured included time to procedure measured in days, adenoma detection rate (ADR), advanced ADR (AADR), adenomas per colonoscopy, sessile serrated polyp detection rate, cecal intubation rate, bowel preparation quality, and compliance with guideline recommendations for surveillance. Patient comorbidities were also recorded. Variable associations with adenoma detection and compliance with surveillance guidelines were analyzed with univariate and multivariate logistic regression.

**RESULTS:**

In total, 235 veterans (mean age, 64.6 years, and 95.7% male) underwent a CCC and had an appropriately matched VAC. ADR in the community was 36.9% compared with 62.6% for the VAC group (*P* < 0.0001). The mean number of adenomas per procedure in the community was 0.77 compared with 1.83 per VAC (*P* < 0.0001). CCC AADR was 8.9% compared with 18.3% for VAC (*P* = 0.003). The cecal intubation rate for community colonoscopies was 90.6% compared with 95.3% for VA colonoscopies (*P* = 0.047). Community care compliance with surveillance guidelines was 74.9% compared with 93.3% for VA (*P* < 0.0001). This nonconformity was primarily due to recommending a shorter interval follow-up in the CCC group (15.3%) compared with the VAC group (5.5%) (*P* = 0.0012). The mean time to procedure was 58.4 days (±33.7) for CCC compared with 83.8 days (±38.6) for VAC (*P* < 0.0001). In multivariate regression, CCC was associated with lower ADR (odds ratio 0.39; 95% confidence interval, 0.20–0.63) and lower compliance with surveillance guidelines (odds ratio 0.21; 95% confidence interval, 0.09–0.45) (*P* < 0.0001 for both).

**DISCUSSION:**

Time to colonoscopy was significantly shorter for CCC compared with VAC. However, compared with VA colonoscopies, there was significantly lower ADR, AADR, and surveillance guideline compliance for services rendered by community providers. This impact on quality of care should be further studied to ensure that colonoscopy quality standards for veterans are not compromised by the process of care and site of care.

## INTRODUCTION

The Veterans Access, Choice, and Accountability Act of 2014 expands the number of options veterans have for receiving care to ensure that veterans have timely access to high-quality care, including colon cancer screening and surveillance. This has been further reformed most recently as the Veterans Affairs (VA) Maintaining Internal Systems and Strengthening Integrated Outside Networks Act of 2018, which instituted an updated elective community care program (1). With the recent implementation of the VA Maintaining Internal Systems and Strengthening Integrated Outside Networks Act, veterans' ability to receive care outside of the VA is expanding. It is paramount to recognize any ensuing variance in patterns of care in the VA vs community settings to ensure that healthcare quality is not compromised in the process. Discrepancies in quality of care have been previously described in regional studies and call into question the pervasiveness of similar trends elsewhere (2). Colonoscopy quality metrics, such as adenoma detection rate (ADR), have been widely accepted and demonstrated to be one of the strongest predictors of interval colon cancer after screening colonoscopy (3). A higher mean number of adenomas per colonoscopy is correlated with higher ADR in respective endoscopists and previously been proposed as a quality indicator worthy of incorporating into associated quality processes as well (4). Substantial evidence exemplifies the marked decreases in colorectal cancer mortality and incidence that correspond to increases in ADR (5). Hence, ADR is considered a robust quality measure and incorporated into quality benchmarks, as reflected in the Multi-Society Task Force guidelines for screening and surveillance colonoscopy (6–8). Surveillance colonoscopy in patients with prior adenomas has been demonstrated to decrease colorectal cancer incidence (9). Thus, proper compliance with surveillance guidelines is critical to interrupt colonic neoplasia development. Alternatively, the overutilization of surveillance colonoscopy can be considered equally problematic, resulting in excess costs and unnecessary risk exposure, without a proven health benefit. Optimizing quality performance measures and resource utilization is essential to improving patient outcomes. Feedback interventions in colonoscopy from quality monitoring and reporting do result in substantial performance improvements in ADR and other quality indicators for the corresponding endoscopists (10). However, there are minimal data currently available analyzing the impact and quality of colonoscopy metrics in veterans receiving procedures within the VA system vs referral to community settings. Given the recent changes in the process of care for veterans created by laws designed to improve access, our goal was to examine the impact of these changes on colonoscopy quality metrics and surveillance recommendations.

## METHODS

### Patient inclusion/exclusion criteria

**Figure 1.**
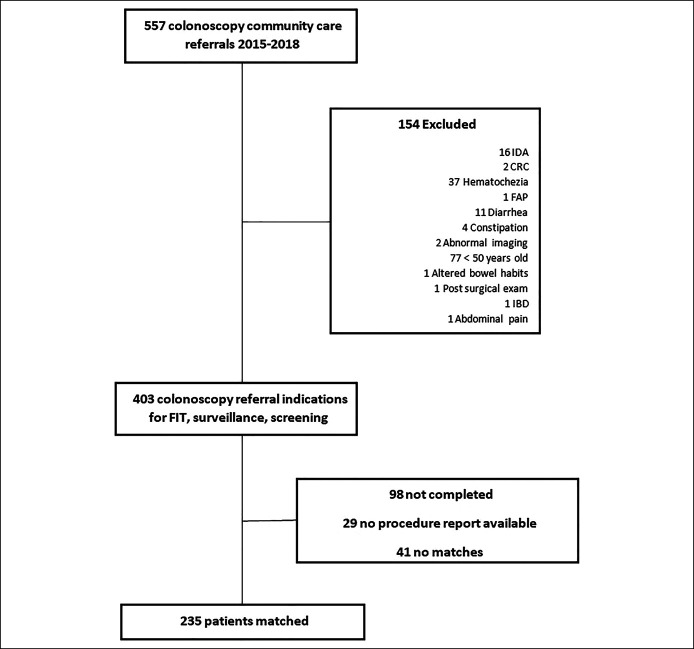
Community care colonoscopy cohort derivation. CRC, colorectal cancer; EGD, esophagogastroduodenoscopy; FAP, familial adenomatous polyposis; FIT, fecal immunochemical test; IBD, inflammatory bowel disease; IDA, iron-deficiency anemia.

**Figure 2.**
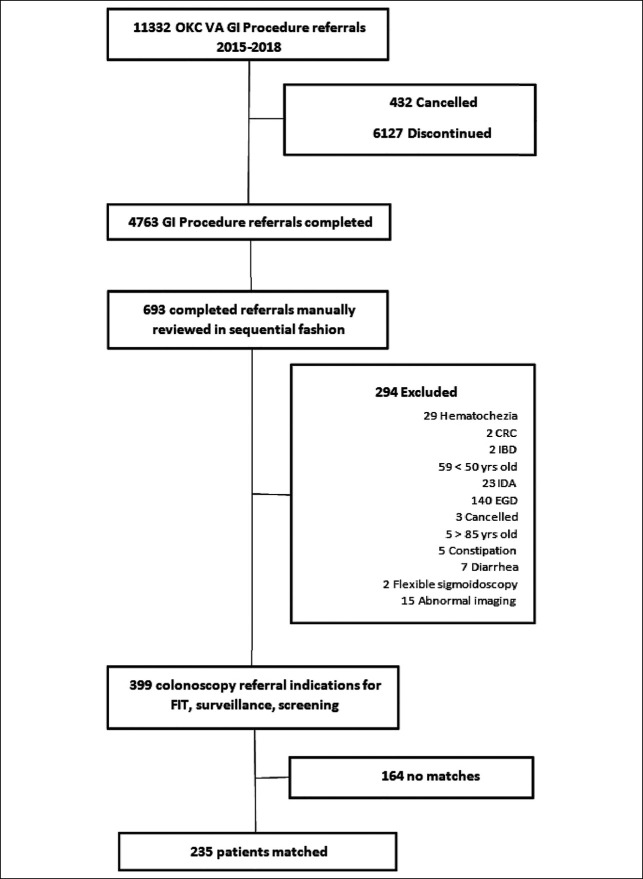
VA colonoscopy cohort derivation. CRC, colorectal cancer; EGD, esophagogastroduodenoscopy; FIT, fecal immunochemical test; GI, gastrointestinal; IBD, inflammatory bowel disease; IDA, iron-deficiency anemia; OKC VA, Oklahoma City VA Medical Center; VA, Veterans' Affairs.

All Oklahoma City VA Medical Center patients aged 50–85 years who were referred for colonoscopy for the indication of colorectal cancer screening, adenoma surveillance, or positive fecal immunochemical test (FIT) results from 2015 to 2018 were eligible for inclusion. Patients who underwent diagnostic colonoscopy for the indication of diarrhea, hematochezia, melena, Crohn's disease, or ulcerative colitis were excluded. Data were extracted into a deidentified database and then reviewed in a sequential fashion. The methodology to generate derived cohorts is reflected in (Figures 1 and 2). Veterans who received colonoscopy at community centers (CCC) were matched for sex, age at time of procedure, and year of procedure in a 1:1 fashion with patients who received a VA colonoscopy (VAC) for the same inclusion indications. Referral to CCC was based on legislative requirements (i.e., geographic proximity [>40 miles] and time to procedure [>28 days until specialty care on request]) (11). Patients and controls were identified without knowledge of the procedure findings (Table 1).

**Table 1.** Baseline characteristics

	VA (n = 235)	Community care (n = 235)	*P*
Age, yr, mean ± SD	64.6 ± 6.3	64.6 ± 6.3	1.00
Male, n (%)	225 (95.7)	225 (95.7)	0.83
Screening, n (%)	53 (22.6)	53 (22.6)	1.00
Surveillance n, (%)	119 (50.6)	97 (41.3)	0.04
FIT, n (%)	63 (26.8)	86 (36.6)	0.02
Performed by nongastroenterologist, n (%)	21 (8.9)	24 (10.2)	0.64
DM, n (%)	81 (34.5)	102 (43.4)	0.047
Obesity, n (%)	131 (55.7)	110 (46.8)	0.05
Smoking, n (%)	125 (53.2)	166 (70.6)	<0.0001
FHX of CRC, n (%)	31 (13.2)	24 (10.2)	0.32
Adequate bowel preparation, n (%)	221/235 (94)	96/142 (67.7)	<0.0001
Quality reported, n (%)	235 (100)	142 (60.4)	<0.0001
Cecal intubation rate, n (%)	224 (95.3)	213 (90.6)	0.047
Pathology reported, n (%)	196 (84.9)	116 (49.4)	<0.0001
Surveillance documented, n (%)	225 (95.7)	163 (69.4)	<0.0001
Surveillance appropriate, n (%)	210/225 (93.3)	122/163 (74.9)	<0.0001
Time to procedure, median (IQR)	49.5 (36–71)	83.0 (57–109)	<0.0001

DM, diabetes mellitus; FHX of CRC, family history of colorectal cancer (first or second degree); FIT, fecal immunochemical test; IQR, interquartile range; VA, Veterans' Affairs.

### Data collection and outcomes of interest

Approval for the study was granted by the Institutional Review Board at the University of Oklahoma Health Sciences Center and the Oklahoma City VA Research and Development Committee. Data were extracted through retrospective chart review by the investigators (V.P. and E.T.) and recorded for both the VAC and CCC groups. The pertinent results of interest included the percentage of community care referrals completed, bowel preparation quality, time to procedure from the time of consult placed (days), pathology results, adenomas detected, cecal intubation rate, recommendations for surveillance, and observation of the established surveillance guidelines. Patient comorbidities were also recorded. Pathology documentation was defined as correspondence to the patient and referring provider recorded in the electronic medical record. VA electronic medical records were reviewed manually to determine if the pathology results were recorded and conveyed to the provider or patient, including addendums to procedure notes, clinic notes, telephone encounters, and scanned documents. Procedure reports were manually reviewed to yield a polyp count per procedure and correlated with pathology reports from the procedure. Reports containing the templates of proprietary software as recognized by the reviewers were considered to be derived from endoscopy software. Quality metrics, including ADR, adenoma per colonoscopy (APC), and adenomas per positive colonoscopy (APPC), were calculated for each group. Advanced ADR was also recorded, with advanced adenomas defined as any adenoma greater than or equal to 10 mm or histology containing cancer/high-grade dysplasia or a significant villous component. Using colonoscopy findings and procedure indication, the 2012 US Multi-Society Task Force guidelines (current at the time of data extraction) were applied to ascertain that recommendations were compliant with appropriate surveillance interval (12). Procedures that had follow-up recommendations provided or available were included for analysis for compliance with the surveillance guidelines. If no recommendations for the follow-up were provided, it was omitted from analysis although recorded for reference as noted in Table 1. Analysis of adequacy of bowel preparation was limited to procedures that had bowel preparation quality reported in procedure documentation. If no bowel preparation quality was reported, it was omitted from the aforementioned analysis. If no bowel preparation quality was reported, but surveillance recommendations were made, the procedure was included in surveillance recommendation analysis and treated as if the preparation quality was adequate. The cecal intubation rates included all procedures, regardless of the quality of bowel preparation.

### Statistical methods and analysis

Continuous and categorical variables of matched populations were analyzed using the *t* test and χ^2^ test, respectively. Variable associations with adenoma detection and compliance with surveillance guidelines were analyzed by univariate analysis. Variables with *P* values <0.1 in univariate analysis were then included in a multivariate logistic regression model using SAS (version 9.4; SAS Institute, Cary, NC) statistical software to generate odds ratios (ORs). Significant values were reviewed to ensure that all variables were included in the multivariate model. Time to procedure in mean days was tested with univariate analysis revealing skewed distribution. The Mann-Whitney test was then conducted using time to procedure in median days with interquartile range and demonstrated statistical significance.

## RESULTS

### Patient baseline demographics

From 2015 to 2018, a total of 235 veterans (mean age, 64.6 years, and 95.7% male) underwent a CCC and had an appropriately matched VA colonoscopy. Procedures in the CCC group were provided by 30 community providers with no academic or gastrointestinal fellowship training centers involved, and all VAC procedures were performed by 1 of 9 academic gastroenterologists or 1 colorectal surgeon. Most of the VAC group had trainees present. Comparisons of the 2 groups are outlined in Table 1. Of the VA procedures, there was a slightly higher percentage of surveillance because the indication compared with community-performed procedures (50.6% VA vs 41.3% community; *P* = 0.04). Community procedures had a slight propensity toward FIT as an indication (26.8% VA vs 36.6% community; *P* = 0.02). Screening indications were identical between both groups (22.6%). Surgeons performed 8.9% of the VA colonoscopies compared with 10.2% for the community setting, which was not statistically significant (*P* = 0.64).

Patients who received care at VA facilities were more likely to be obese, diabetic, have adequate bowel preparation, documented pathology results, a higher cecal intubation rate, and the quality of examination and recommendations recorded in procedure notes. Patients receiving care in the community were more likely to have a history of tobacco smoking. The mean time to VA colonoscopy was approximately 25 days longer than a community colonoscopy (83.8 ± 38.6 days VA vs 58.4 ± 33.7 days community; *P* < 0.0001).

### Adenoma detection rate

ADR was significantly lower with community facilities compared with the VA facility cohort (36.9% CCC vs 62.6% VAC; *P* < 0.0001). Similarly, the mean number of APC in the community was lower (0.77 vs 1.83; *P* < 0.0001) compared with those performed within the VA system (Table 2). The mean number of APPC was lower in the community compared within the VA (2.10 ± 1.72 CCC vs 2.94 ± 3.02 VAC; *P* = 0.0189). The same disparity was found regarding advanced ADR (8.9% vs 18.3%; *P* = 0.003), and sessile serrated polyp detection rate (3.4% vs 7.7%; *P* = 0.044) for procedures performed within the community care compared with the VA, respectively.

**Table 2.** Polyp detection

Variable	VA	Community care	*P*
Adenoma detected (ADR%)	147 (62.6)	86 (36.7)	<0.0001
Mean polyps per procedure ± SD	3.59 ± 3.9	1.44 ± 2.1	<0.0001
Mean no. of adenomas per procedure	1.83 ± 2.8	0.77 ± 2.8	<0.0001
Mean no. of APPC	2.93 ± 3.02	2.10 ± 1.72	0.0189
Advanced ADR (%)	45 (19.1)	21 (8.9)	0.0015
SSPDR (%)	18 (7.7)	8 (3.4)	0.044
HGD/CA (%)	4 (1.7)	2 (0.85)	0.412

ADR, adenoma detection rate; APPC, adenoma per positive colonoscopy; HGD/CA, high-grade dysplasia/carcinoma; SSPDR, sessile serrated polyp detection rate; VA, Veteran's Affairs.

There was no statistically significant difference in ADR between gastroenterologist-performed and nongastroenterologist-performed procedures (Table 3). In multivariate regression analysis (Table 4), CCC was independently associated with a lower ADR (OR 0.39, 95% confidence interval [CI], 0.2–0.63; *P* < 0.0001). Cecal intubation (4.0, 95% CI, 1.2–13.3; *P* = 0.02) and history of diabetes (OR 1.86, 95% CI, 1.22.9; *P* = 0.006) were associated with an increased ADR (Table 3). Despite the observation, adequate bowel preparation was significantly associated with ADR in univariate analysis, and this association was not significant in the multivariate regression analysis.

**Table 3.** Adenoma detection

Variable	Adenoma detected (n = 233)	No adenoma detected (n = 237)	*P*
Age, yr, mean ± SD	65.0 ± 6.1	64.3 ± 6.5	0.18
Male sex, n (%)	223 (95.7)	226 (95.4)	0.85
Obesity, n (%)	112 (48.1)	129 (54.4)	0.17
Smoking, n (%)	144 (61.8)	147 (62.0)	0.96
DM, n (%)	109 (36.8)	74 (31.2)	0.0005
FHX of CRC, n (%)	30 (12.9)	25 (10.6)	0.43
Screening, n (%)	55 (23.6)	53 (22.6)	1.00
Surveillance, n (%)	106 (45.5)	110 (46.4)	0.84
FIT, n (%)	72 (30.9)	77 (32.5)	0.71
Surgeons, n (%)	19 (8.2)	26 (10.9)	0.29
Adequate preparation, n (%)	181/199 (90.6)	136/178 (76.4)	<0.0001
Community care, n (%)	86 (36.9)	149 (62.9)	<0.0001
Cecal intubation rate, n (%)	228 (97.8)	209 (88.2)	<0.0001

DM, diabetes mellitus; FHX of CRC, family history of colorectal cancer (first or second degree); FIT, fecal immunochemical test.

**Table 4.** Multivariate associations with adenoma detection rate

Associations with adenoma detection rate	Multivariate		
Variable	Odds ratio	95% confidence interval	*P*
Community care	0.39	0.25–0.63	<0.0001
Diabetes mellitus	1.86	1.2–2.9	0.006
Preparation quality adequate	1.57	0.81–3.06	0.19
Cecal intubation	4.0	1.2–13.3	0.02

### Surveillance guideline compliance

Surveillance recommendations were documented for 163 CCC patients (69.4%) and 225 VAC patients (97.8%) (*P* < 0.0001) (Table 1). Community care compliance with the surveillance guidelines was 74.9% (122/163) compared with 93.3% (210/225) for VA (*P* < 0.0001) (Tables 1 and 4). This nonconformity was primarily due to recommending a shorter interval follow-up in the CCC group compared with the VA colonoscopy group (15.3% vs 5.5%; *P* = 0.0012). Longer intervals than guideline recommendations were also more common in the community setting as opposed to the VA (9.8% vs 0.8%; *P* = 0.0001). In multivariate regression analysis, CCC was independently associated with lower compliance with the surveillance guidelines (OR 0.21, 95% CI, 0.09–0.45; *P* < 0.0001) (Table 5). Although nongastroenterologist-performed procedures, adequate bowel preparations, and adenoma detected during examination were all factors significantly associated with guideline noncompliance in univariate analysis, they were not significant in the multivariate regression analysis (Table 6).

**Table 5.** Compliance with surveillance guidelines

Variable	Appropriate surveillance recommendations (N = 332)	Inappropriate surveillance recommendations (N = 56)	*P*
Age, yr, mean ± SD	65.1 ± 5.8	64.5 ± 6.4	0.50
Male, n (%)	318 (95.8)	51 (91.1)	0.13
Screening, n (%)	79 (23.8)	6 (10.7)	0.03
Surveillance n, (%)	150 (45.2)	34 (60.7)	0.03
FIT, n (%)	104 (31.3)	16 (28.6)	0.68
Surgeon, n (%)	33 (9.9)	1 (1.8)	0.045
Obesity, n (%)	180 (54.2)	24 (42.9)	0.12
Smoking, n (%)	199 (59.9)	39 (69.6)	0.17
DM, n (%)	138 (41.6)	21 (37.5)	0.57
FHX of CRC, n (%)	41 (12.4)	4 (7.1)	0.26
Adequate bowel preparation, n (%)	260/293 (88.7)	31/44 (70.5)	0.001
Adenoma detected, n (%)	180 (54.2)	22 (39.3)	0.039
Endoscopic software used, n (%)	120 (36.3)	22 (39.3)	0.66
Community care, n (%)	122 (36.8)	41 (73.2)	<0.0001
VAC, n (%)	210 (63.2)	15 (26.8)	<0.0001

DM, diabetes mellitus; FHX of CRC, family history of colorectal cancer (first or second degree); FIT, fecal immunochemical test; IQR, interquartile range; VAC, VA colonoscopy.

**Table 6.** Multivariate associations with compliance with surveillance guidelines

Associations with compliance with surveillance guidelines	Multivariate		
Variable	Odds ratio	95% CI	*P*
Adenoma detected	1.18	0.56–36.42	0.03
Community care	0.21	0.09–0.45	<0.0001
Performed by nongastroenterologist	4.54	0.56–36.42	0.15
Screening indication	1.40	0.48–4.11	0.54
Surveillance indication	0.41	0.18–0.91	0.03
Adequate bowel preparation	1.74	0.74–4.09	0.21

## DISCUSSION

We observed a pronounced difference in adenoma, advanced adenoma, sessile serrated lesion, and adenomas detected per colonoscopy between VA and community settings. The absolute difference in ADR was over 25%. Because previous studies have suggested a strong correlation of ADR with future colorectal cancer, the significant difference in quality has the potential to strongly influence future colorectal cancer outcomes, including death. One study found a decrease of 1% in the ADR results in a 3% increase in interval cancer risk and a 5% increase in the risk of cancer mortality (5). Our findings are congruent with previous regional studies revealing a discrepancy in colonoscopy quality metrics, most prominently ADR, between VA and non-VA providers (2). This is further compounded by the antecedent observation of higher colorectal neoplasia prevalence in US veterans compared with the civilian population, although recent data have produced conflicting results (13). In addition, we found a significantly higher mean APC for patients receiving a VA colonoscopy, comparable with other VA cohorts. The total number of APC has been proposed as a superior metric more accurately identifying high-performing endoscopists and quality colonoscopy performance compared with ADR to avoid the one-and-done phenomenon that might artificially increase ADR as a true measure of comprehensive mucosal inspection (14). Although the retrospective nature of this study creates some uncertainty of the true APC, because of the imprecise correlation of multiple polyps in 1 specimen container and final pathology results, the consistency of this metric with other metrics suggests that the difference is real. Although a divergence in ADR between gastroenterologists and nongastroenterologists has been observed elsewhere, this was not observed in our study (2). The small number of procedures done by nongastroenterologists may have limited our ability to detect any differences, but international studies suggest that this specialty difference in quality may be a US-based phenomenon if, in fact, present at all (15,16). Indeed, the large number of community providers used for CCC and the heterogenous nature of reports preclude meaningful analysis of the quality metrics of individual endoscopists in this study.

We also found that surveillance recommendations were less frequently provided in the community, and when they were available, were less likely to be compliant with the surveillance guidelines compared with VA-performed procedures. We suspect varying competency with the technical skills of adenoma detection correlates with situational awareness at time of examination and the application of appropriate interval follow-up. It is also possible that the isolated care event created by the community referral mechanisms within VA health care alter the communication of recommendations for care needed several years later. Lack of compliance with the surveillance guidelines has also been found in previous studies. Departure from accepted guidelines has previously resulted in the overuse of colonoscopy in one-quarter of patients with a history of low-risk adenomas/no adenomas and underuse or no colonoscopy at all in nearly half of VA the patients with a history of high-risk adenomas (17). These trends have also been noted to be widespread not only in VA settings but also in non-VA settings and with a larger impact in the community setting (18,19). As a response, a nationwide VA initiative for colon cancer screening and surveillance clinical reminders has been implemented, and some VA hospitals have rolled out their own clinical decision support system for monitoring the postcolonoscopy patient follow-up and scheduling to ensure compliance with the surveillance guidelines (20,21). Such interventions are needed to optimize resource utilization for colorectal neoplasia surveillance and when community care procedures lack guideline-driven recommendations the value of these efforts is compromised.

Recent data suggest that the impact of surveillance colonoscopy correlates strongly with decreased risk of interval colorectal cancer long after the index examination, making appropriate timely follow-up critical, and any significant deviations thereof, worthy of close scrutiny. In a recent colon cancer screening trial, a large cohort of nearly 3000 patients were found to have high-risk adenoma findings on initial screening colonoscopy. Over one-third of these patients for whom a 3-year surveillance examination was recommended did not complete a follow-up colonoscopy within 5 years (9). Yet, another study of 3 large, distinguished US academic centers including over 6,000 patients with advanced adenomas at first screening colonoscopy revealed widely varying and suboptimal adherence to surveillance intervals. At best, approximately 30% compliance for surveillance at certain centers was recorded, and at worst, as low as only 10.2% (22). Clearly, efforts to curb colon cancer rates using surrogate outcomes, such as advanced adenoma detection, suffer greatly when colonoscopy screening and surveillance are not optimized, and making initial recommendations for correct surveillance intervals is the first step in this care pathway.

Why there was such a radical difference in ADR observed in our study between VA and non-VA providers is unclear but is consistent with patterns previously noted (2,14). Wang et al. noted a similar discrepancy concerning the total number of adenomas in veterans detected between tertiary care academic center colonoscopy vs nonacademic-provided colonoscopy. Advanced ADR was much higher in the VA center compared with contracted community facilities as well. Bartel et al. found comparable ADR inequalities between CCC and VA-provided colonoscopy as well, with community ADR 38% neatly approximating our observed result of 36%, whereas VA provided examinations excelled above 50% in both studies. There are likely multiple influences, including the desperate healthcare systems, involved in community care, including heterogenous communication mechanisms with veterans, regarding procedure preparation. Ensuring quality is not compromised when veterans are referred to the community should be a priority. Discrepancy for the quality of care is not unique to gastroenterology services. Recent literature has demonstrated worse community outcomes in other specialties as well, such as cardiology, where even increased mortality has been observed in cardiac procedures conducted outside the VA compared with within the VA (23). Although we did not collect data on the extent or nature of trainee involvement in VA-provided colonoscopies, the University of Oklahoma is an academic affiliate of the Oklahoma City VA where fellow and surgical resident participation is a routine occurrence. A portion of the ADR variance may be related to trainee involvement, which has been associated with increased ADR in other similar studies as well (24–26). Looking at other quality measures, although bowel preparation was not independently associated with ADR in our small cohort, it remains possible that it was a contributing force to the differences observed and has been detected to be in other studies (27). Society guidelines recommend achieving an adequate bowel preparation in >85% of the procedures (8). This metric was missed in the community cohort and exceeded in the VA colonoscopy cohort. Similarly, the time pressures in community practices may alter the time allocated to colonoscopies relative to VA procedures. These time pressures and the lack of continuity of care inherent in the community care procedural referrals may compromise the quality of procedures referred to the community. Although there was no mechanism to compare withdrawal times between the CCC and the VAC groups because of the lack of reporting, the compromised ADR in the CCC group strongly suggests less thorough mucosal inspection. Furthermore, one-and-done is an endoscopic approach speculated to be an unintended consequence of the ADR dichotomy that makes no distinction for the total number of lesions identified and successfully resected. ADR is a quality benchmark that can readily be attained and credited on an isolated finding. It has been speculated that reimbursement schemes create perverse incentives to encourage a one-and-done approach in the community, given there is minimal to no additional financial reward for additional polypectomy performed beyond the initial resection (28). The higher rate of APC and APPC in the VAC group relative to the ADR increase in the VAC vs CCC group confirms that the overall quantity of adenomas removed was higher in the VAC group. Both latter measures have been proposed as a more precise quality measure to help overcome these negative incentives to colonoscopy quality (14). Finally, the isolated care event created by the community referral process may lead to less than consistent follow-up and investment in longitudinal patient care by providers, which is evident in the lower rate of documented patient communication of results in the CCC group. The time pressures and variable care settings in community settings may also be factors contributing to the lower compliance with the surveillance guidelines. Although financial incentives might explain shorter interval recommendations, approximately 40% of the noncompliant recommendations were for longer intervals. This suggests lack of familiarity with current guidelines in a significant percentage of providers participating in these procedures. Given the competing forces of resource utilization and cancer prevention provided by colonoscopy and polypectomy, optimizing the timing of surveillance examinations is important to achieve high-value outcomes. Regretfully, inconsistent procedure documentation, incomplete results communication, and discordant follow-up recommendations for surveillance repeat colonoscopy remain rampant, which recently has been studied and all demonstrated to be significant contributors to interval postcolonoscopy cancer (29). The importance of adherence to quality measures has been illustrated in the latest iteration of the American College of Gastroenterology guidelines (30), which has stressed strong quality improvement and monitoring programs to be instrumental to reducing postcolonoscopy colorectal cancer. Such quality indicators recommended to be closely followed include not only ADR and withdrawal time, but also cecal intubation goal of >95%, which was exceeded in the VA cohort but not the community care cohort, and also associated with ADR in multivariate analysis (30). We did note a longer average wait until procedure for VAC, approximately 25 days longer until examination compared with CCC. Although timeliness is very important, a slightly longer time to examination on the scale of several weeks is of marginal consequence. Indeed, recent data suggest higher risk of colorectal cancer, and advanced disease is not incurred when colonoscopy is delayed for up to 6 months but even as long as 10 months after a positive fecal occult blood test (31,32). A slight delay to achieve substantial gains in quality and colonoscopy outcomes should be viewed as a favorable trade off.

Like other retrospective studies, a limitation of our study includes the possibility of selection bias. This was curtailed as much as the study design permitted by matching both cohorts according to year of examination performance, sex, and age of patients. The substantial magnitude of absolute difference in primary outcomes between well-matched groups would be difficult to wholly ascribe to selection bias. There were minor patient characteristic differences between the cohorts. There were more diabetics in the VA cohort. Diabetes was associated with an increased ADR in our study, and it is a previously described risk factor for increased development of colonic adenomas, and it is possible that this may have exacerbated the difference in ADR between the groups (33). However, risk factors, such as obesity and smoking that are also expected to be associated with an increase in colon neoplasia, were more prevalent in the community cohort. Because the 2 cohorts derived from the same VA population pool, similarities in the patient profile are to be expected and further diminish the threat of unaccounted confounders undermining or amplifying the observed results. Despite matching, we could not control for incomplete, inconsistent, or even absent reporting from community providers concerning bowel preparation and surveillance interval recommendations. Community care procedures fell well short of the recommend bowel preparation documentation frequency of >98% (8). Given nearly one-third of community procedures did not document any recommendations at all, this may more accurately reflect a broader underlying decreased proficiency among community providers rather than a reporting bias predisposing to potentially undetected effects in the community group. Although the frequency of screening colonoscopy was identical between the groups, VA procedures were more likely to have a history of previous adenomatous polyps and performed for surveillance compared with community care procedures. This has the potential to increased adenoma prevalence in the VAC cohort. However, a recent study also examining ADR at VA hospitals in these indications similarly revealed an ADR of 50% when pooling nonscreening indications and found no statistically significant difference when compared with screening indications, further emphasizing that slight heterogeneity in indications is unlikely to skew our results (34). Similarly, our results did not note a difference in ADR between screening, positive FIT, and surveillance examination indications. Finally, the proportion of patients referred to community centers with an adequately matched control is a small fraction of all VA colonoscopy referrals, limiting our analysis to a smaller sample size, but based on the statistical size of the differences in the groups, it was adequately powered. Another limitation of our study is the limited information regarding community care physicians performing colonoscopy. Owing to the large number of providers and heterogenous nature of reports received, it was not possible in this analysis to assess quality metrics at the individual level. It is unclear whether any were routinely monitoring quality metrics, such as ADR, bowel preparation reporting, cecal intubation rates, and minimum withdrawal time. A lack of having these measures reported back to the providers may limit their performance. This lack of provider quality reporting also limits the veteran's access to information they may find useful when deciding to pursue community vs VA-based care. More importantly, when the veteran returns to the VA for continuity of care, the referring primary care provider and the patient frequently had no provisional guidance on when the next interval surveillance colonoscopy is indicated potentially compromising the veteran's care.

Our research suggests that previous findings in the Northeastern United States are not isolated to regional variances but, in fact, far more widespread than previously believed and sets the stage for further community and VA studies on a national scale. It is abundantly evident that the substantial disparity in the detection rate of neoplastic polyps and the technical examination performance strongly influence primary colonoscopy quality outcomes, which are well-established predictors of future colorectal cancer incidence and outcomes. Efforts focused on minimizing wide variation of colonoscopy quality are needed to narrow currently existing gaps in the community and hence mitigate the future risk of incident colorectal cancer. As aforementioned, the VA closely monitors colonoscopy quality and is subject to a national Veterans Health Administration health directive governing implementation and patient outcome parameters within VA facilities (20). It may be worthwhile to take measures to ensure that community referrals are held to the same gastrointestinal society and Veterans Health Administration standards expected within the VA health system. Although the Veterans Access, Choice, and Accountability Act was enacted to improve the timeliness of care for veterans, the possible unintended consequences on the quality or consistency of care warrant investigation. Our study clearly demonstrates some compromises in the quality of care in patients referred for CCC that have the potential to far exceed any benefits of improved timeliness of care. Policy makers, administrators, and other stakeholders should be aware of these dynamics because they make decisions regarding the allocation of resources. For the benefit of veterans' health, it may improve outcomes to focus on VA facility access or have quality metric accountability for community providers. The results of our study and previous studies of colonoscopy quality suggest that further studies of other veteran health outcomes in the community vs Department of Veterans Affairs facilities are needed.

## CONFLICTS OF INTEREST

**Guarantor of the article:** William M. Tierney, MD.

**Specific author contributions:** V.P.: collected, interpreted, and analyzed data, and drafted and revised the manuscript. E.T.: collected data. M.M.: planned study, analyzed data, and performed statistical analysis. W.M.T.: planned and supervised the study, interpreted data, drafted, and revised the manuscript. All authors approve final draft submitted.

**Financial support:** None to report.

**Potential competing interests:** None to report.

**Study Highlights**

**WHAT IS KNOWN**

✓ Federal law permits eligible veterans to seek care outside of the Department of Veterans' Affairs (VA) setting according to geographic and time constraints.
✓ Many veterans receive colonoscopy outside of the VA.

**WHAT IS NEW HERE**

✓ Non-VA colonoscopy was associated with shorter wait to procedure.
✓ Non-VA colonoscopy had a lower adenoma detection rate.
✓ There was lower compliance with the surveillance guidelines outside of the VA.
